# Within-host viral dynamics of dengue serotype 1 infection

**DOI:** 10.1098/rsif.2014.0094

**Published:** 2014-07-06

**Authors:** Hannah E. Clapham, Vianney Tricou, Nguyen Van Vinh Chau, Cameron P. Simmons, Neil M. Ferguson

**Affiliations:** 1 Department for Infectious Disease Epidemiology, MRC Centre for Outbreak Analysis and Modelling, Imperial College, London W2 1PG, UK; 2 Institut Pasteur de Bangui, Bangui, Central African Republic; 3 Hospital for Tropical Diseases, District 5, Ho Chi Minh City, Vietnam; 4 Oxford University Clinical Research Unit, University of Oxford, District 5, Ho Chi Minh City, Vietnam; 5 Centre for Tropical Medicine, Nuffield Department of Medicine, University of Oxford, Oxford OX1 2JD, UK; 6 Nossal Institute for Global Health, University of Melbourne, Melbourne, Victoria 3010, Australia

**Keywords:** dengue, virus dynamics, within-host modelling

## Abstract

Dengue, the most common mosquito-borne viral infection of humans, is endemic across much of the world, including much of tropical Asia and is increasing in its geographical range. Here, we present a mathematical model of dengue virus dynamics within infected individuals, detailing the interaction between virus and a simple immune response. We fit this model to measurements of plasma viral titre from cases of primary and secondary DENV 1 infection in Vietnam. We show that variation in model parameters governing the immune response is sufficient to create the observed variation in virus dynamics between individuals. Estimating model parameter values, we find parameter differences between primary and secondary cases consistent with the theory of antibody-dependent enhancement (namely enhanced rates of viral entry to target cells in secondary cases). Finally, we use our model to examine the potential impact of an antiviral drug on the within-host dynamics of dengue. We conclude that the impact of antiviral therapy on virus dynamics is likely to be limited if therapy is only started at the onset of symptoms, owing to the typically late stage of viral pathogenesis reached by the time symptoms are manifested and thus treatment is started.

## 1. Introduction

Dengue, the most common arboviral disease of humans, is endemic across much of tropical Asia, Latin America and possibly parts of Africa [1]. Recent global estimates suggest approximately 400 million infections per year resulting in approximately 100 million apparent illnesses [1]. There are four dengue virus types (DENV 1–4), and each is capable of causing clinical disease. Human infection is acute and self-limiting, with a wide spectrum of disease severity that ranges from a mild undifferentiated illness to severe and life-threatening dengue shock syndrome [2].

Primary infection with one of the four dengue serotypes (DENV 1–4) is thought to lead to lifelong immunity to that serotype, but in addition to generate a temporary period of cross-protective immunity to all serotypes [3,4]. However, subsequent infection with a heterologous serotype is more likely to result in severe disease than primary infection [5–11]. The mechanism for this is not fully understood, but a leading hypothesis is antibody-dependent enhancement (ADE), whereby antibodies generated in the primary infection are not sufficient to neutralize the virus, but still attach to the virus particles and, as neutralized virus would be, are taken up by cells such as macrophages. Unlike virus bound to neutralizing antibody, virus bound to non-neutralizing antibody is capable of infecting macrophages, amplifying the viral replication process [12]. A role is also hypothesized for ‘original antigenic sin’, in which there is preferential activation of memory T or B cells with lower than optimal avidity for the infecting virus [13,14].

The mechanisms underlying the transient vascular permeability syndrome that is a feature of severe dengue are as yet not clear, but high viraemia levels early in the illness have been implicated in some studies [15–18], though not in others [19]. Various mechanisms secondary to high viral burdens are speculated to account for the vascular permeability syndrome, though none have been validated in either animal models or by clinical interventions. Differences in the humoral immune response between primary and secondary DENV infections have been observed, with cross-reactive antibodies [20,21] dominating the response in a secondary infection, and differences in the length of viraemia have also been noted [16].

The target cell population for DENV replication is not well characterized. *In vitro*, monocytes [22–24], dendritic cells [23], endothelial and epithelial cells among others were found to support replication. However, it is not clear how these findings relate to *in vivo* infection. Given the systemic nature of human DENV infection, it is reasonable to assume a variety of cell types and tissues are infected *in vivo*.

Elucidating the dynamics of dengue pathogenesis is useful to help understand the mechanisms of infection and for the rational development of interventions such as antivirals or vaccines. Though there are currently no dengue antivirals available, development is ongoing and there have been early antiviral trials (for example the data in this paper come from a trial of chloroquine treatment) [25]. Evaluating how antivirals might modify the within-host dynamics of dengue infection in the context of treatment only starting at the earliest with the onset of symptoms (and therefore relatively late in infection) is of use for the rational development and application of antivirals.

In addition, as viral dynamics affect infectiousness of an infected host to a mosquito taking a blood meal, better characterization of virus dynamics is relevant to understanding transmission. This has applications for vector-control strategies targeting vector competence, the most notable example being the use of the bacterial symbiont *Wolbachia* to infect *Aedes aegypti*, where the level of transmission blocking induced by *Wolbachia* infection depends on the human viral titre when the mosquito feeds [26].

Mathematical modelling of the interaction between the virus and immune response, validated against available quantitative data on viral kinetics, has proved a powerful tool for gaining such understanding in other infections. For example, in a set of seminal papers Ho, Perelson, Neumann and co-workers [27,28] examined HIV dynamics under therapy, elucidating important virus properties such as the lifespan of infected cells and virus. More recently, models of acute infections have been developed, including influenza [29–34] and measles [35].

Little modelling of within-host dengue pathogenesis has been undertaken previously. A statistical mechanics approach was used to explore the immune response to dengue vaccination [36], while other work considered a simple dynamical model of virus and immune dynamics [37], but did not examine alternative modes of immune action, the difference between primary and secondary disease, and did not fit the model to data. Most recently, another theoretical study of potential differences in within-host viral dynamics between primary and secondary infection has been published, but was not linked to individual patient data throughout infection [38]. Here, we develop a mathematical model of dengue pathogenesis which includes a simple representation of the clearing immune response. We use the model to characterize the viral dynamics of both primary and secondary dengue infections by fitting to DENV 1 viral titre data measured at multiple time points throughout infection from a large number of patients with clinically apparent dengue infection. The resulting parameter estimates allow us to hypothesize as to the factors that could be governing the heterogeneity observed in infection dynamics between individuals infected with the same serotype (DENV 1) and between primary and secondary DENV 1 cases.

## 2. Material and methods

### 2.1. Data

The data used to parametrize the model were derived from a clinical trial of chloroquine in adult dengue patients at the Hospital for Tropical Diseases in Ho Chi Minh City, Vietnam, by Tricou *et al*. [39]. There were no significant differences found between the placebo and treatment groups in this original clinical trial of choloroquine treatment [39]. The treatment and placebo groups results have previously been analysed together [40] and we do likewise here. Blood was taken twice daily from arrival in hospital for a minimum of 5 days and RT-PCR was used to quantify virus RNA in plasma; measurements are per millilitre of plasma. The assay used either had a limit of detection (LOD) of 1500 copies ml^−1^ or 15 000 copies ml^−1^. Both infectious and non-infectious virions are detected using this assay.

**Figure 1.**
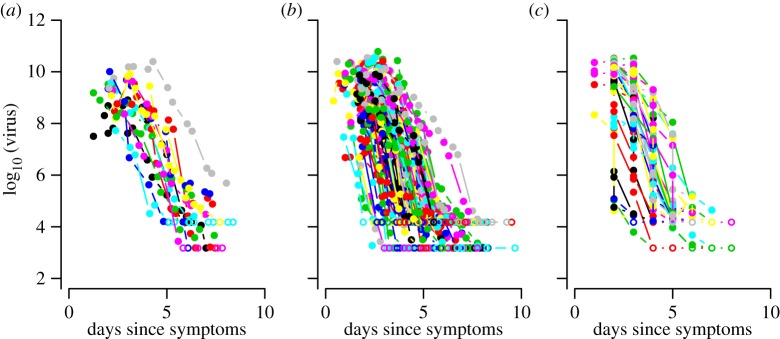
Plot of viral load data from hospitalized dengue patients used in this study. Filled points are viral load measurements above the LOD; unfilled points show measurements below the LOD (*a*) primary DF, (*b*) secondary DF and (*c*) secondary DHF.

We use individual patient data on DENV-1 primary dengue fever (DF, *n* = 15), secondary DF (*n* = 91) and secondary dengue haemorrhagic fever (DHF, *n* = 32) (figure 1). See source paper for details on classifications [40]. There were not enough primary DHF patients in these dataset for statistically significant conclusions to be drawn (*n* = 3), so we do not use those data for model fitting (primary DHF data are shown in the electronic supplementary material, figure S1).

Viraemia measurements are reported at various time points following reported symptom onset and always begin within 72 h of reported symptom onset. A full description and analysis of the data can be found in the papers published on the source study [39,40].

### 2.2. Model definition

Given the limited data available, we use one of the simplest models of virus and immune dynamics [41]. This model has four state variables: the population sizes of free virus (*v*), uninfected target cells (*x*), infected target cells (*y*) and an (adaptive) clearing immune response (*z*). Free virus infects target cells via a mass-action process with rate *β*, and infected cells produce more virus at rate *ω*. Using a single-state variable for the immune response is clearly a gross simplification, but in the absence of detailed data on correlates of immunity in acute dengue to fit the model to, a more complex representation cannot be robustly parametrized. The (deterministic) model is defined by the following ordinary differential equations:
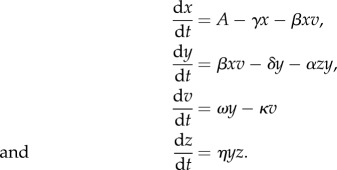


Target cells are produced at a constant rate throughout infection (*A*) and have a mean lifespan 1/*γ*. Infected cells have a lifespan in the absence of the immune response of 1/*δ*. The lifespan of free virus is 1/*κ*. The model is very similar to that used previously for a theoretical study of dengue within-host dynamics [37].

This model assumes the immune response proliferates in response to infected cells at rate *η*, with a decay rate which is assumed negligible over the timescale of dengue infection. The initial size of the immune response population is *z*_0_. Immunity acts by clearing infected cells (again via a mass action process) at rate *α*.

The basic reproduction number (mean number of infected cells produced by each infected cell at the start of infection), *R*_0_, for this model, is given by *R*_0_ = *β x*_0_ *ω*/*κ*(*δ* + *αz*_0_), where *x*_0_ is the initial number of target cells, and the other parameters as defined above.

As we appreciate this is only one possible model of immune action, we also consider a model variant in which the immune response acts by clearing free virus instead of infected cells (see the electronic supplementary material, equations S1). In the absence of additional data, we do not currently consider a more complex immune response or target cell model.

### 2.3. Parameter estimation

Table 1 lists all model parameters, states whether they are estimated or assigned, and, if assigned, their default values (with references). Estimated parameters can be fitted as patient-specific, group-specific or common to all patients (global). All assigned parameters are global. We consider three patient groups: primary DF, secondary DF and secondary DHF cases.

**Table 1.** Parameters of the model and their values if assigned.

parameter	description	estimated or assigned	value if set
*γ*	uninfected cell death rate per day	assigned (global)	0.14 (7 day mean lifespan) [42]
*A*	target cell production per millilitre per day	assigned (global)	1.4 × 10^6^, 1.4 × 10^7^ (giving target cell densities of 10^7^ or 10^8^ ml^−1^) [43]
*β*	infection rate of target cells per virion (includes proportion of virions that are infectious and the rate of entering target cells)	estimated (global or group)	—
*α*	removal rate of infected cells per immune cell per day	assigned (global)	0.001 (arbitrary-scales with *z*_0_)
*δ*	baseline infected cell death rate per day	assigned (global)	0.14 (7 day mean lifespan) [42]
*ω*	production rate of virions per infected cell per day	assigned (global)	1 × 10^4^ [44]
*κ*	virion clearance rate per day	estimated (global or group)	—
*η*	proliferation rate of immune cells per infected cell per day	estimated (global, group or patient)	—
*z*_0_	initial population size of immune effector population per millilitre	estimated (global, group or patient)	constrained less than 1 to ensure immune response is not initially shaping dynamics
*v*_0_	initial inoculum of virus per millilitre	assigned (global)	1
IP	incubation period	estimated (patient)	prior from: [45,46]

We assign certain parameters because, in the absence of data on the size of the target cell or immune effector populations, not all parameters are independent. Substituting *x*′ = *x*/*A*, *y*′ = *y*/*A*, *v*′ = *βν* and *z*′ = *αz* in the differential equations above demonstrates that out of *η*, *ω* and *A*, only *ω A* and *η A* can be estimated independently, and that similarly only *α z*_0_ can be estimated, not *α* and *z*_0_ independently (see the electronic supplementary material, equations S2). In addition, estimates of *ω* and *β* are expected to be inversely correlated. We therefore do not fit the parameters *A*, *ω*, *α* and instead assign values to these parameters for all patients. The first two are set to plausible values, and the third (arbitrarily) to 0.001/day.

In addition, the excess death rate of infected cells proved difficult to resolve given the much larger impact of immune-related clearance of infected cells. We therefore assumed infection did not shorten the life of target cells except via the action of the immune response.

Assigned parameter values were taken from the literature (table 1), and we also explored sensitivity analyses to assess what impact these assumed values have on the other estimated parameter values. For target cell numbers, the density of monocytes lies in the standard range 0.2–0.8 × 10^6^ ml^−1^ blood [43] or 0.36–1.5 × 10^6^ ml^−1^ plasma (assuming 55% of blood is plasma). We explore different target cell densities up to 10^8^ ml^−1^ of plasma, as monocytes represent only a small fraction of all macrophages, with most macrophage populations being distributed in other body tissues, and much virus replication thought to occur in these tissue-based cells.

We assign values of the rate of virus production per infected cell, *ω*, using data from *in vitro* experiments in which virus output from infected cells was measured [44]. We discuss the sensitivity to the values assumed later in this paper.

Our baseline assumption was that target cells had a mean life of 7 days, comparable to estimates for activated macrophages [42], but also examine the effect of assuming much longer lived target cells (mean life of 2 years). For the equilibrium density of target cells to remain fixed, varying the target cell death rate, *γ*, requires the rate of target cell recruitment, *A*, to be scaled proportionately, so examining a scenario of long-lived target cells is equivalent to exploring the effect of very low target cell replenishment rates.

We then estimate the remaining parameters. We used Markov chain Monte Carlo (MCMC) methods in a Bayesian framework for parameter estimation [47]. Code was written in R [48] and C. Parameters were updated singly using the Metropolis–Hasting algorithm, with the median and 95% credible intervals for the parameters reported. A burn-in of 300 000 updates was used and then a 1 in 100 sample of the following 700 000 updates used to calculate posterior distributions. MCMC traces of each parameter were plotted and convergence was assessed visually; runs where all parameters converged were accepted. To assess differences between patient groups in the estimated values of patient-specific parameters, the joint posterior distributions across all patients within a group were compared.

The viral titre data we analyse are reported as a function of time since self-reported symptom onset. However, we wished to model from the beginning of infection, so it was necessary to estimate the incubation period (IP) or equivalently, the time of infection, which we would expect to be correlated with the (unobserved) initial virus inoculum, *v*_0_. The viral inoculum may differ between people depending on the level of virus inoculated by a mosquito when it bites and whether it is interrupted during feeding. In the models here, we fixed *v*_0_ at a value of 1 copy ml^−1^ plasma, and then estimated the IP. Using data from previous infection experiments [45,46], we assigned a normal prior on the IP with a mean of 5.7 days and s.d. of 1.73 days. The priors on all other parameters were kept vague, using improper flat uniform distributions.

To fit to the data, we assumed log viraemia measurements had normally distributed errors. For measurements below the LOD, we need to take into account the fact that all we know about these measurements is that the viraemia is at or below the LOD. This means we need to use the cumulative distribution function (cdf) of the likelihood density in the likelihood. In the likelihood expression below, *φ* and *ϕ* are, respectively, the probability and cumulative density functions (pdf and cdf) of the normal distribution, *n* is the numbers of observations, *D_i_* are the viraemia measurements and *x_i_* are the model predictions. *σ*^2^ was taken to be 1. Following Howey *et al*. [49], for the measurements under the LOD, *c_i_* = 0 if *D_i_* > LOD (value was 1500 or 15 000 depending on the PCR assay used) and *c_i_* = 1 if not.
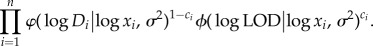


Log-likelihood values (together with a qualitative assessment of model fit) were used to assess how well each model variant recreated patient and group-level variation in virus dynamics.

In order to see which factors best explained the observed variation in viral kinetics between patients and groups of patients (i.e. primary DF versus secondary DF versus secondary DHF cases), we considered a variety of model variants with fitted parameters being estimated on one of three levels: patient-specific, group-specific or global. The six models variants considered and the parameters estimated at each level for each are shown in table 2.

**Table 2.** Table of the six models, the parameters in each model estimated at each of the three levels and the mean posterior log-likelihoods values for the different model variants considered.

model number	global parameters	group-specific parameters	patient-specific parameters	median log-likelihood
1	*κ*, *β*	—	*z*_0_, *η*, IP	−981
2	*κ*, *β*	*z*_0_	*η*, IP	−1085
3	*κ*, *β*	*η*	*z*_0_, IP	−2235
4^a^	—	*κ*, *β*	*z*_0_, *η*, IP	−857
5	—	*z*_0_, *κ*, *β*	*η*, IP	−947
6	—	*η*, *κ*, *β*	*z*_0_, IP	−1139

^a^All with target cell density 10^7^ (shown in figure 3).

We assumed *a priori* that it would be biologically plausible for (some of) the immune response parameters and the IP parameter to vary between patients, and then considered the extent to which variation in each of these immune response parameters was able to recreate the variation observed between individuals. Preliminary fitting (not shown) demonstrated that it was necessary for the IP parameter to be patient-specific. However, if the IP was assumed to be the only patient-specific parameter, it was not possible to reproduce the observed between-patient variation in virus dynamics. Model variants assuming different combinations of parameters fitted as patient-specific are described in models 1–3 (table 2). Models 2 and 3 take each of the immune-related parameters assumed to be patient-specific in model 1 and assess the loss of model fit seen when the variation in that parameter was assumed to be at group level rather than at the individual patient level. We then examined how model fit improved if the virus parameter fitted as global in models 1–3 were fitted as varying by patient group (table 2, models 4–6).

There may be differences in fitness between different dengue 1 virus genotypes [44,50], but as the cases analysed in this paper arose from one short-time period, we did not consider this further here.

### 2.4. Modelling antiviral treatment

We used our model to consider the potential impact of antiviral treatment on virus dynamics. The model used to simulate an antiviral treatment that completely blocks virus production is shown below.
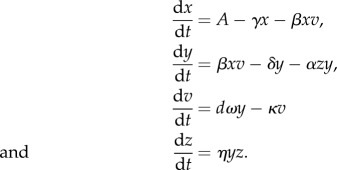


State variables and parameters are as in the main model, with the addition of *d*, where *d* = 0 after drug administered, and 1 before. We assume that antiviral treatment begins immediately after a patient is admitted to hospital. We do not model the pharmacokinetics or dynamics of drug, and in the absence of data otherwise, assume that the drug is above therapeutic levels immediately and remains so for the remainder of infection.

Using parameter estimates from the posterior distributions, we evaluate the effect of this modelled antiviral treatment on viral kinetics for the primary DF, secondary DF and secondary DHF patient groups separately, using the following three summary measures: percentage change in the area under the complete log_10_ viral titre curve (AUC) (setting all modelled titres under the LOD (=1500) to 1500 and then subtracting log_10_(1500) from each log_10_ titre measurement), log_10_ change in virus peak titre and change in number of days until viral titre reaches the LOD (1500 ml^−1^).

## 3. Results

**Figure 2.**
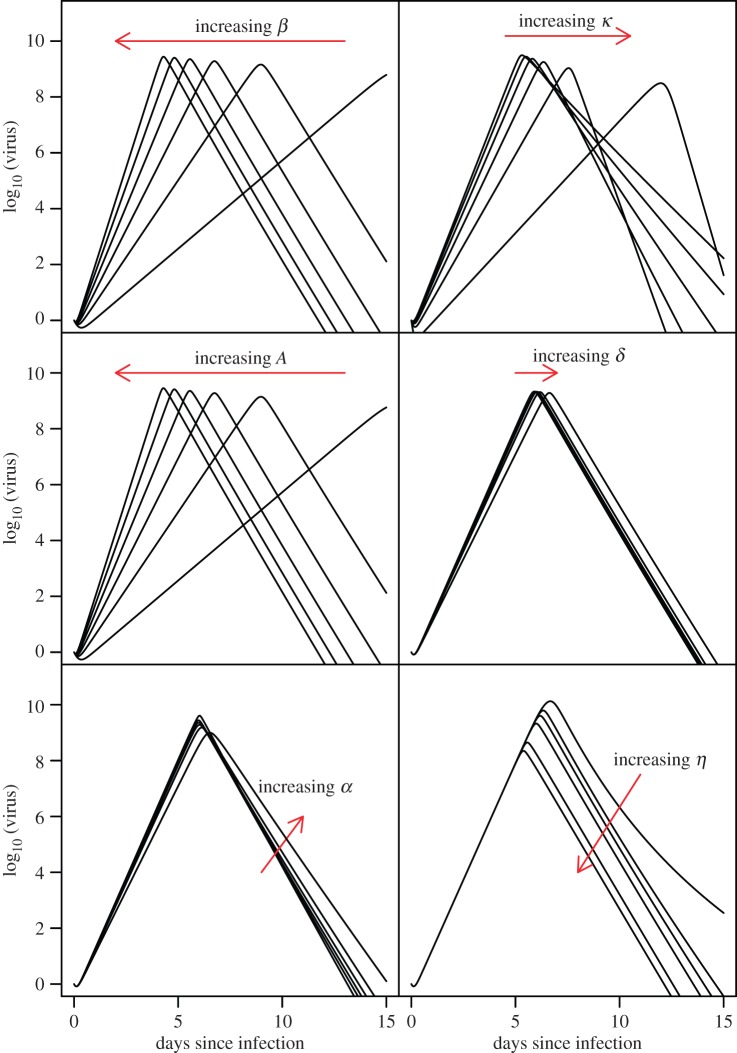
Sensitivity of model dynamics to parameters. Viral dynamics shown for *R*_0_ values of 20, 30, 40, 50, 60 and 70 by varying (from top left to bottom right) parameters *β*, *κ*, *A*, *α* and *δ* οne at a time given baseline parameter values (which result in *R*_0_ ≈ 35) of *β* = 3 × 10^–10^, *α* = 0.1, *ω* = 10 000, *η* = 10^−5^, *κ* = 3.5, *δ* = 0.14, *γ* = 0.14, *A* = 1.4 × 10^6^. *η* (which does not affect *R*_0_) was varied with the following values: 10^−6^, 3 × 10^−6^, 5 × 10^−6^, 10^−5^, 5 × 10^−5^, 1 × 10^−4^ and 5 × 10^−4^.

Figure 2 shows the sensitivity of viral dynamics to model parameters, varying one parameter at a time. Except for *η* (which does not affect *R*_0_), we vary each parameter so as to step through the following set of values of *R*_0_: 20, 30, 40, 50, 60 and 70. This range was selected to span the values of *R*_0_ estimated by fitting the model to patient data (see below). As would be expected from the expression for *R*_0_ given in the Material and methods, we see that increasing *β* (virus entry to cell) or *A* (target cell production) increases *R*_0_ and viral growth rate and causes earlier peaking of virus. Increasing *κ* (virus clearance rate) reduces *R*_0_ and viral growth rate, leading to virus peaking later and increases the virus clearance rate. Increasing *η* (immunity proliferation rate) also moves the peak earlier by achieving earlier control of virus, but with no impact on initial viral growth rates. Changes in *α* (immune mediated clearance rate of infected cells) or (equivalently) *z*_0_ (initial size of immune cell population) have a subtle impact in this model: as expected, initial viral growth rates are not substantially affected, but increases in these parameters cause immune control of virus replication to occur when immune cell populations are at a lower level, which can lead to viraemia decaying more slowly thereafter (though this may be sensitive to the assumed form of the immune response proliferation). Model dynamics are relatively insensitive to the value of *δ* (infected cell death rate in the absence of immunity), justifying our choice not to fit this parameter.

**Figure 3.**
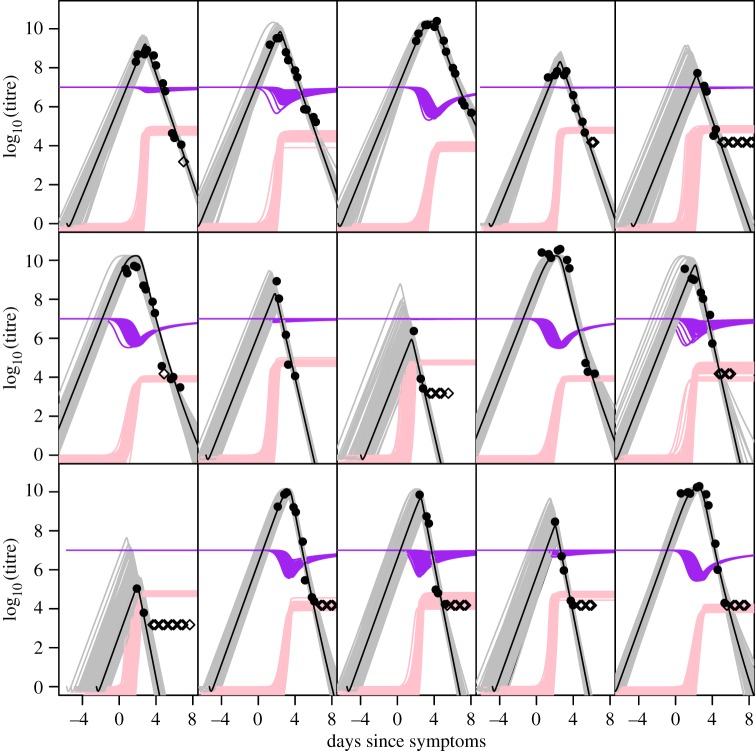
Best fit model (by likelihood); model 4 (with *z*_0_, *η*, IP fitted as patient-specific and *β*, *κ* fitted as group-specific) fitted to all patients, assuming *A =* 1.4 × 10^6^ ml^−1^ d^−1^. Results shown for representative selected patients, all outputs on a log scale. Viral load data points shown as black dots (filled: above detection limit; unfilled: below the LOD). Black lines, fitted median viral dynamics; grey lines, sample from posterior for virus dynamics; pink lines, sample from posterior for immune response dynamics; purple lines, sample from posterior of target (uninfected) cell dynamics. First row, primary DF patients; second row, secondary DF patients; third row, secondary DHF patients.

Table 2 lists the model fits undertaken and their likelihoods, which vary by which parameters were fitted on a global, patient- or group-specific basis. Predicted viral dynamics of the model variant which gave the best fit (in terms of likelihood) (fitting *z*_0_, *η* and IP as patient-specific parameters and *β*, *κ* as group-specific: model 4) is shown for five patients from each patient group in figure 3. Computed immune response and target cell dynamics are also shown.

Allowing *η* and IP to vary between patients (models 2 and 5) is enough to recreate the differences we see between individuals’ dynamics in viral peak height and timing, but cannot produce variation in initial virus growth rate. Owing to the lack of data on the early stage of infection (prior to symptoms), we know little about much of the variation that occurs between individuals in this period. The addition of variation in *z*_0_ between patients improves the fit, but varying this parameter alone (with the IP—models 3 and 6) cannot recreate the variation in peak viral titre and timing observed. Allowing for variation in *z*_0_ in addition to *η* and IP (model 1) substantially increases the log-likelihood (models 1 and 4), though formally not sufficiently to justify statistically the large number of parameters added. However, in qualitative terms, this model does produce more reasonable, less sharp virus peaks, and hence we present results for model 4 henceforth. Estimated parameter values for this model are shown in table 3 and in the electronic supplementary material, table S1, for the other models.

**Table 3.** Parameter estimates for the *κ*, *β* group-specific, *z*_0_, *η*, IP patient-specific model (model 4, (likelihood in table 2), all assuming a target cell density (*A*/*γ*) of 10^7^ ml^−1^). Patient-specific parameter estimates are summarized by taking the median of each posterior was taken and the median of these medians is shown for each patient, inter-quartile range (in curved parentheses) and maximum/minimum (in squared parentheses) obtained across all fitted patients. Medians and 95% credible intervals are shown for the common and group-specific parameters. Model fit shown in figure 3.

model	parameter	primary DF	secondary DF	secondary DHF
*κ*, *β* group-specific: *z*_0_, *η*, IP patient-specific × model 4	*β*(×10^−10^)	1.72 (1.51, 2.04)	2.30 (2.17, 2.43)	2.82 (2.62, 3.01)
	*κ*	3.48 (3.30, 3.67)	5.29 (5.08, 5.52)	6.07 (5.71, 6.43)
	*z*_0_	0.347 (0.326, 0.402) [0.285, 0.548]	0.411 (0.338, 0.466) [0.248, 0.594]	0.380 (0.326, 0.439) [0.0298, 0.526]
	*η*	1.29 × 10^−5^ (8.86 × 10^−6^, 6.24 × 10^−5^) [7.96 × 10^−7^, 1.09 × 10^−3^]	2.95 × 10^−5^ (6.77 × 10^−6^, 9.48 × 10^−5^) [7.36 × 10^−7^, 3.95 × 10^−3^]	2.71 × 10^−6^ (9.48 × 10^−7^, 8.07 × 10^−5^) [5.01 × 10^−7^, 0.224]
	incubation period (IP)	5.80 (5.48, 6.33) [4.79, 6.76]	5.77 (5.42, 6.02) [4.54, 8.29]	5.69 (5.09, 6.72) [1.72, 9.32]

We examined the sensitivity of model fit to the assumed density of target cells (controlled by the parameter *A*). In general, for fixed *ω*, increasing the density of target cells improved model fit, allowing the model to reproduce peaks viral titres seen in some secondary DF patients that are missed when assuming a lower target cell density (see the electronic supplementary material, table S2). However, if *ω* (viral production rate per infected cell per day) is allowed to increase by the same factor as *A* is decreased by, then the quality of fit can be maintained for lower values of *A*, though the resulting values of *ω* quickly become unrealistically large.

In order to assess what is driving the differences observed in primary and secondary virus dynamics, it is informative to compare parameter estimates between DENV1 primary (DF) and secondary (DF and DHF) case patient groups. Patient-specific variation in *z*_0_, *η* and IP was not sufficient alone to capture group differences (table 2, model 1). Allowing virus parameters to vary between case groups (table 2, model 4) produced a better likelihood and generated a model in which estimates of viral transmission rates (*β*) and virus killing rates (*κ*) were higher for secondary cases than for primary cases. These values are consistent with the theory of ADE and are consistent across models 4–6. In model 4, these parameters are greater for the secondary DHF group than the secondary DF group, though this is not consistent across all model variants. Interestingly, despite these parameter differences, *R*_0_ values are similar across groups: the mean *R*_0_ value is 35 (95% CI: 29, 40) for primary DF, 30 for secondary DF (95% CI: 27, 34) and 33 for secondary DHF (95% CI: 29, 37).

**Figure 4.**
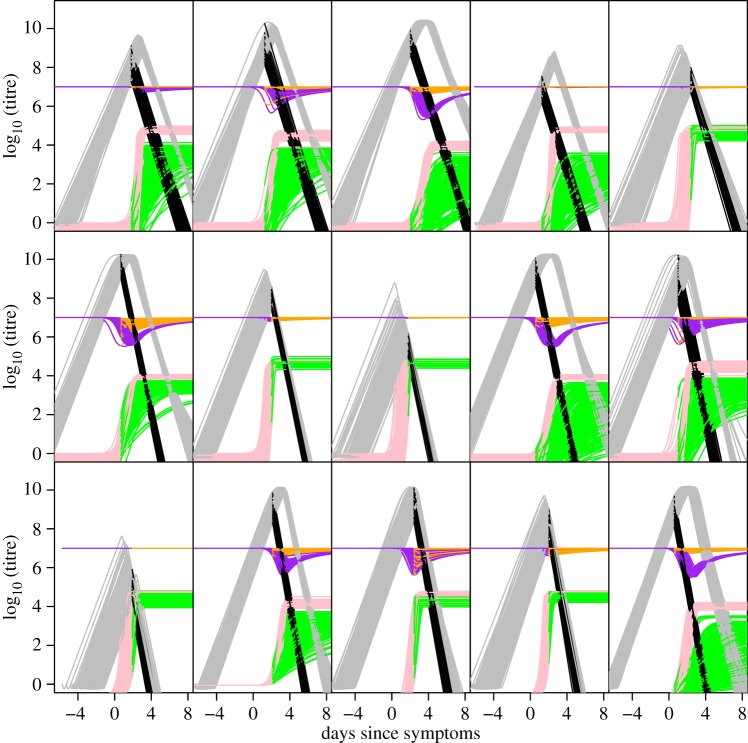
Impact of antivirals on virus dynamics, same selected patients as above. Model dynamics as figure 3 shown with the addition of the considered antivirals. Sample from posterior of dynamics of virus without antiviral are in grey. Virus dynamics with an antiviral are shown in black. Target cell dynamics without antiviral are shown in purple and with antiviral are shown in orange. Immune response dynamics without antiviral are shown in pink and with antiviral are shown in green.

Using the model variant with the highest likelihood (table 2, model 4), we examined the potential impact of antiviral treatment on viral dynamics, summarized using a number of measures (table 4 and figure 4). It should be noted that antiviral treatment would only be administered after onset of symptoms and therefore after a substantial fraction of viral replication would have occurred, limiting the potential impact on virus dynamics. The previously highlighted heterogeneity in virus dynamics between individuals is also important here, with antiviral treatment estimated to have considerable impact on some patients, but a negligible impact for patients arriving at hospital at a later stage of infection. Antivirals have a somewhat greater impact in primary versus secondary cases, due to peak viraemia being more likely to occur after arrival in hospital (and therefore after treatment onset) in primary cases (though it is rare even in that patient group) and because rates of viral decline in the absence of treatment are higher in secondary infection compared with primary [40]. Antivirals in this model have a negligible impact on peak virus titres.

**Table 4.** Impact of antiviral treatment initiated immediately after onset of symptoms for a drug which reduces viral production from infected cells. The model was run for each patient in our dataset with the 200 samples from the posterior distributions for the parameters of the best fit model (by likelihood, model 4—*z*_0_, *η*, IP fitted as patient-specific and *β*, *κ* fitted as group-specific), without the antiviral (as shown in figure 4) and with the antiviral (administered on arrival to hospital and active immediately). Three measures of antiviral impact were then compared per sample from the posterior distributions with and without the antiviral, and differences averaged across all samples in a patient group. Mean and 95% credible interval reported. Virus was assumed undetectable at less than 1500 copies ml^−1^.

	primary DF	secondary DF	secondary DHF
average % change in log_10_ virus AUC	−9.96 (−22.8, −1.76)	−7.68 (−21.8, −0.845)	−7.58 (−20.6, −1.05)
average difference in log_10_ peak virus	−0.633 (−1.49, 0.0101)	−0.402 (−1.72, 0.0157)	−0.362 (−1.45, 0.0133)
change in days until virus undetectable	−1.64 (−4.62, −0.247)	−1.74 (−4.64, −0.191)	−1.73 (−4.79, −0.213)

## 4. Discussion

We have presented a simple mathematical model of dengue virus dynamics within a human host, and by fitting to data from DENV1 patients, estimated model parameters. By allowing parameters to vary by patient or patient group, we were able to capture the observed heterogeneity between patients and between primary and secondary infection.

We found it was necessary to vary the immune response proliferation rate (*η*) and the IP between people to reproduce the variation in viral kinetics seen (the majority of which is seen in timing and magnitude of peak titres). This provides evidence for a key role of the immune response proliferation in shaping virus dynamics, supporting the conclusions of earlier theoretical work [37].

We found relatively high target cell densities (10^6^–10^7^ for each ml of plasma) were required for the model to reproduce observed viral dynamics while keeping viral production rates per infected cell at reasonable levels [44]. This is in keeping with tissue reservoirs playing an important role in pathogenesis, with virus produced in such reservoirs contributing substantially to viraemia seen in plasma. We find some evidence to support the role of target cell depletion in shaping virus dynamics, with more rounded peaks in viraemia being consistent with those predicted by our model when target cell depletion is significant. However, this conclusion must remain tentative until more data are available, in particular to allow the impact of infection on target cell lifetime to be estimated.

Previous analysis of the dataset we use here found some evidence for DENV1 secondary infections being of shorter duration than DENV1 primary infections, with secondary cases more likely to arrive at hospital at or after peak virus titre and having a faster rate of virus decline [40]. This is despite the fact that primary and secondary cases were assessed to not arrive on significantly different days of symptoms. While these trends are not clear-cut, our analyses reproduce these differences via some systematic differences in parameter estimates between primary and secondary infection groups.

When considering differences between primary and secondary cases, the best fitting model we found had the virus clearance rate (*κ*) and rate of cell entry (*β*) varying by patient group. This model predicts a greater value of *β* in secondary cases, a finding consistent with the theory of ADE whereby antibody aids virus entrance to cells in a secondary infection. This model also predicts that *κ* is greater in secondary cases (with some suggestion that *η* is also larger in secondary infection), consistent with an increased activation of the immune system in secondary infection and therefore faster viral clearance. We find few significant differences between the DF and DHF secondary infection patient groups, but slight differences are seen in one model variant which might suggest even higher values of *β* for DHF cases.

We tested whether our results were robust to uncertainties in assigned parameter values and model specification. In the electronic supplementary material, table S3, figures S2*a* and S2*b*, we show that the differences between patient groups hold for the model in which the immune response clears virus instead of infected cells, strengthening this still tentative conclusion. This result is also insensitive to the assumed rate of the target cells regeneration during infection (see the electronic supplementary material, table S4 and figure S3).

A shortcoming of our analysis is the absence of data on viral titres in the early stages of infection (before symptoms) and how early viral kinetics vary between primary and secondary cases (as noted, we rarely see the peak of viraemia in primary cases and even more rarely in secondary cases). Reducing the numbers of parameters being estimated (e.g. by fitting *η* as a group-level parameter—table 2, model 6) constrains model dynamics, resulting in viral peaks being inferred as occurring before the first data point. However, even with this more constrained model, we still find the same systematic differences in the estimates of other parameters between primary and secondary infections (see the electronic supplementary material, table S1).

Obtaining viral titre data early in infection, and considering it in the context of these models, would clearly improve our understanding of dengue pathogenesis, but such data are challenging to collect. Two possible sources are household studies (where blood samples are taken from members of households in which an index case has been detected) and human challenge studies, should the latter receive ethical approval.

Our analysis has a number of other limitations, principal among which is the fact we are solely analysing viral titre data [51]. The absence of data on the target cell population or effector immune response necessarily limits model complexity with the assumptions about both necessarily needing to be kept simple. One result of the simple representation of the immune response used is that the model predicts an early increase and plateau in immune response and sharp peaks in viraemia in the absence of substantial target cell depletion. This, together with the absence of data on target cell populations, means our conclusions about target cell population sizes and depletion are necessarily tentative. With additional data, it may be possible to parametrize more complex models of the immune response, the different impacts of the innate and adaptive immune response and to include explicitly how immune responses are modified in secondary infection.

The main mechanism by which the immune response controls infection is of importance for understanding how virus is cleared, how viral clearance is modified during secondary infection and how it could be modulated by antiviral drugs. In our results, we are not definitively able to distinguish between the two forms of action we considered (clearance of infected cells and of free virus). For the results presented above, we assumed that the immune response clears infected cells. We found broadly similar results (notably for the differences in parameter estimates between primary and secondary infection) for a model in which the immune response is assumed to directly clear free virus instead. However, in this alternative model our estimates of the intrinsic virus life span are very short (a few hours), meaning any immune response targeting free virus needs to act within minutes of the virus being released from an infected cell to give a reduction in the within-host *R*_0_ sufficient to achieve control of infection. The extent to which such fast clearance is biologically feasible is open to question, though this could be possible with an effective antibody. Infected cells are expected to have a considerably longer lifespan for other infections [28,29], giving more opportunity for immune clearance to act, though this would also depend on the extent to which virus production destroys cells.

In our model, the impact of antiviral treatment on virus dynamics of secondary DENV1 cases is predicted to be less than on primary DENV1 cases on average, because cases arrive in hospital at a later stage of viraemia in secondary DENV1 infection (despite the day of reported symptoms being similar) and because the rate of decay of virus titre is seen to be higher in secondary cases. There is substantial variation between patients in the predicted impact of antivirals; in some cases, we predict an antiviral would have a minimal impact, while the impact in those treated earlier in infection dynamics is much greater. All this should also be borne in mind for testing antivirals on primary and secondary cases (and possibly, by extension, on different serotypes). Our analysis supports the use of the change in the AUC of log_10_ viraemia as a sensitive measure of antiviral effect on viral dynamics [25], but whether this is the important measure for predicting impact on symptoms is less clear; there may be other more important predictors of symptom severity and duration, such as the time spent above a certain virus titre threshold.

In summary, we have fitted a dynamical model to primary and secondary DENV1 dengue infection viraemia data, with the resulting parameter estimates confirming a role for the immune response in shaping variation between individuals in viral kinetics and generating parameter differences between primary and secondary cases, which are consistent with the hypothesis of ADE. In addition, we considered the possible impact of an antiviral therapy with different modes of action, which should be of use in the development and testing of antivirals. More comprehensive data on both viral titres and immune responses would allow more sophisticated models of dengue pathogenesis to be developed. Other extensions to this work would be to consider viraemia data for different serotypes and from less severe cases, ideally including asymptomatic infections, to fit to data on immune responses and/or target cell populations as well as viral titre and to refine the simple immune response model used (e.g. refining the form of immune proliferation, explicitly modelling the impacts of innate and adaptive immunity and mechanistically representing the differences (e.g. ADE) in immune responses between primary and secondary infection).

## Supplementary Material

Supplementary Information

## Supplementary Material

Data Supplement
