# Supplementary material for: Within-host viral dynamics of dengue serotype 1 infection
Source: J R Soc Interface. 2014 Jul 6;11(96):20140094. doi: 10.1098/rsif.2014.0094 (PMC4032531; doi:10.1098/rsif.2014.0094)

# Within-host viral dynamics of dengue serotype 1 infection

Hannah E. Clapham^1*^, Vianney Tricou^2^, Nguyen Van Vinh Chau^3^, Cameron P. Simmons^4, 5, 6^, and Neil M. Ferguson^1^

1 MRC Centre for Outbreak Analysis and Modelling, Department for Infectious Disease Epidemiology, Imperial College, London, W2 1PG, UK; 2 Institut Pasteur de Bangui, Bangui, Central African Republic; 3 Hospital for Tropical Diseases, District 5, HCMC, Viet Nam; 4 Oxford University Clinical Research Unit, District 5, HCMC, Viet Nam; 5 Centre for Tropical Medicine, Nuffield Department of Medicine, University of

Oxford, Oxford OX1 2JD, UK; 6 Nossal Institute for Global Health, University of Melbourne, VIC 3010, Australia

* Author for correspondence: hannah.clapham08@imperial.ac.uk

**Supplementary Figure S1**

Primary DHF data from the 3 primary DHF DENV1 cases.

**
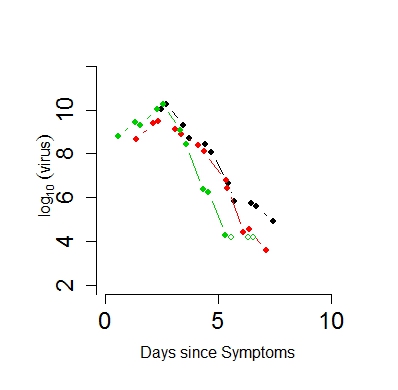
**

**Supplementary Equations S1**

Model variant in which the immune response acts on free virus not infected cells:

$$\begin{matrix} \frac{dx}{dt}=A-\gamma x-\beta xv \\ \frac{dy}{dt}=\beta xv-\delta y \\ \frac{dv}{dt}=\omega y-\kappa v-\alpha zv \\ \frac{dz}{dt}=\eta yz \end{matrix}$$

Parameter* α* now represents the rate at which immunity clears free virus, acting via a mass-action process. Other parameters and all state variables have the same definitions as for the main model.

**Supplementary Equations S2**

Reducing the dimension of the system.

We substitute *x*′=*x*/*A*, *y*′=*y*/*A*, *v*′=*βv*, *z*′=*αz* into the main model equations to give:$\begin{matrix} \frac{dx'}{dt}=1-\gamma x'-x'v' \\ \frac{dy'}{dt}=x'v'-\delta y'-z'y' \\ \frac{dv'}{dt}=\beta\omega Ay'-\kappa v' \\ \frac{dz'}{dt}=\eta\alpha Ay'z' \end{matrix}$

**Supplementary Table S1**

Parameter estimates for the fits of model variants summarised in Table 2 in the main paper. Patient specific parameter estimates are summarized with the median of median parameter estimates for each individual, inter-quartile range (in curved parentheses) and maximum/minimum (in square parentheses) obtained across all fitted patients. Medians and 95% credible intervals are shown for the common and group specific parameters.

| *Model* | *Parameter* | Primary DF | Secondary DF | Secondary DHF |
| --- | --- | --- | --- | --- |
| *κ, β global*  *η, z_0_ IP patient specific*  *Model 1* | *β(x10^-10^)* | 2.23 (2.17, 2.38) | | |
|  | *Κ* | 5.05 (4.83, 5.17) | | |
|  | *z_0_* | 0.549  (0.380, 0.653)  [0.0603 0.931] | 0.392  (0.138, 0.671)  [ 9.23x10^-4^, 0.999] | 0.331  (0.157, 0.701)  [0.00245 0.998] |
|  | *η* | 1.69 x10^-6^  (8.36 x10^-7^, 9.6 x10^-6^)  [4.57x10^-7^ 1.39 x10^-3^] | 2.96 x10^-5^  (2.53x10^-6^, 5.77, x10^-4^)  [4.82 x10^-7^, 4.70] | 9.39 x10^-6^  (2.09 x10^-6^, 1.59 x10^-4^)  [6.37 x10^-7^, 0.263] |
|  | *Incubation Period* | 6.20  (5.46, 6.78)  [3.88 9.36] | 5.82  (5.05, 6.74)  [0.545, 12.1] | 5.97  (5.29, 7.06)  [2.09 8.04] |
| *κ, β global*  *z_0_ group specific*  *η IP patient specific*  *Model 2* | *β(x10^-10^)* | 2.03 (1.95, 2.130) | | |
|  | *κ* | 4.33 (4.23, 4.42) | | |
|  | *z_0_* | 0.774,  (0.244, 0.991) | 7.19 x10^-35^  (4.20x10^-44^, 5.64x10^-27^) | 3.672 x10^-14^  (2.57 x10^-19^ , 4.29 x10^-10^) |
|  | *η* | 6.44 x10^-6^  (3.67x10^-6^, 2.60x10^-5^)  [5.62x10^-7^, 6.79x10^-4^] | 2.88 x10^-5^  (2.32x10^-6^, 5.28 x10^-4^)  [4.00 x10^-6^, 49.9] | 4.49 x10^-5^  (1.00 x10^-5^, 7.20 x10^-4^)  [2.47 x10^-6^, 1.10] |
|  | *Incubation Period* | 5.43  (5.25 , 6.17)  [4.49, 8.26] | 5.62  (4.81, 6.44)  [1.66, 8.43] | 5.99  (5.02, 6.98)  [2.31,7.77] |
| *κ, β global*  *η group specific and then z_0_ IP patient specific*  *Model 3* | *Βx10^-10^* | 1.85 (1.81, 1.97) | | |
|  | *κ* | 4.87 (4.73, 5.13) | | |
|  | *z_0_* | 0.609  (0.287, 0.815)  [0.0917, 0.915] | 0.390  (0.150, 0.670)  [ 0.000359, 0.932] | 0.437  (0.189, 0.762)  [0.00734, 0.999] |
|  | *η* | 5.93x10^-7^  (5.66x10^-7^, 6.77x10^-7^) | 1.78 x10^-6^  (1.25 x10^-6^, 2.17 x10^-6^) | 1.57 x10^-6^  (1.26 x10^-6^, 2.03 x10^-6^) |
|  | *Incubation Period* | 9.76  (9.03 , 10.26)  [7.33, 10.9] | 8.76  (8.04, 9.67)  [4.04x10^-3^, 11.8] | 8.81  (8.44, 9.38)  [7.23, 11.7] |
| *z_0_, κ, β *  *group specific*  *η - IP patient specific*  *Model 5* | *β(x10^-10^)* | 1.71  (1.49, 1.99) | 2.00  (1.88, 2.18) | 3.23  (2.89, 3.62) |
|  | *κ* | 3.37  (3.18, 3.59) | 4.28  (4.20, 4.42) | 5.42 5.78 6.15 |
|  | *z_0_* | 2.72x10^-3^  (9.60x10^-7^, 0.419) | 5.54 x10^-38^  (3.25x10^-44^, 2.47x10^-25^) | 1.52x10^-5^  3.74x10^-8^, 1.79x10^-3^ |
|  | *η* | 2.97x10^-5^  (1.93x10^-5^ 8.77x10^-5^) [1.07x10^-6^, 1.39x10^-3^] | 2.36x10^-4^  (2.28 x10^-5^, 3.35 x10^-3^)  [2.01x10^-6^, 38.5] | 3.72 x10^-6^  (1.11x10^-6^, 9.01x10^-5^)  [5.59x10^-7^, 0.440] |
|  | *Incubation Period* | 5.76  (5.43, 6.18)  [4.80, 6.67] | 5.65  (5.04, 6.53)  [1.67, 11.0] | 5.00  (4.31, 6.13)  [1.99, 10.40] |
| *η, κ, β *  *group specific-*  *z_0_- IP patient specific*  *Model 6* | *β* | 1.85x10^-10^  (1.64, 2.24x10^-10^) | 9.94 x10^-10^  (8.82x10^-10^, 1.11 x10^-9^) | 5.51 x10^-10^  (4.71, 7.90x10^-10^) |
|  | *κ* | 3.40  (3.23, 3.57) | 19.9  (17.4, 22.8) | 9.78  (8.31, 13.6) |
|  | *z_0_* | 0.429  (0.375, 0.495)  [1.12x10^-5^, 0.610] | 0.417  (0.393, 0.441)  [0.00218, 0.548] | 0.418  (0.400, 0.436)  [0.0113 0.485] |
|  | *η* | 4.73x10^-6^  (3.05x10^-6^, 6.98x10^-6^) | 5.56x10^-7^  (5.46x10^-7^, 5.67x10^-7^) | 6.54 x10^-7^  (6.15 x10^-7^, 7.02 x10^-7^) |
|  | *Incubation Period* | 6.35  (5.82, 7.14)  [4.88, 8.02] | 5.53  (4.97, 6.49)  [3.03 8.71] | 5.35  (5.13, 5.88)  [4.08, 8.05] |

**Supplementary Table S2**

Log-likelihood for baseline model fit with 10^6^ target cells.

| **Overall parameters** | **Patient specific**  **Parameters** | **Group specific**  **Parameters** | **Median**  **log likelihood** |
| --- | --- | --- | --- |
| - | *z_0_ η, IP* | *κ, β* | -1056 |

**Supplementary Table S3**

Parameter estimates for the model variant in which the immune response removes free virus (see Supplementary Equations S1 above) assuming a target cell density (*A*/γ) of either 10^7^/ml or 10^8^/ml. Patient specific parameter estimates are summarized with the median of median parameter estimates for each individual, inter-quartile range (in curved parentheses) and maximum/minimum (in square parentheses) obtained across all fitted patients. Medians and 95% credible intervals are shown for the common and group specific parameters (shown in Supplementary Figure S2a and S2b). Parameter *κ *ιs fixed at *3.5* where the value is such that virus particles survive on the order of hours in the absence of immune response as it thought for RNA viruses. *κ *not *δ* is now fixed as the immune response is acting on the virus *κ *will be more difficult to resolve. *ω* is fixed at 1x10^3^ as before *β, δ* are fitted varying at group level,* η, IP* and *z_0_*  are fitted as varying at patient level

| **Model** | **Parameter** | **Primary DF** | **Secondary DF** | **Secondary DHF** |
| --- | --- | --- | --- | --- |
| A/γ=107/ml  Log-likelihood= -1152 | *β(x10^-10^)* | 3.83  (3.50, 4.11) | 5.11  (4.90, 5.31) | 4.98  (4.59, 5.20) |
|  | *δ* | 3.91  (3.64, 4.14) | 5.96  (5.70, 6.37) | 5.63  (5.31, 5.88) |
|  | *z_0_* | 0.392  (0.332, 0.451)  [0.217 0.510] | 0.365  (0.290, 0.409)  [0.025 0.616] | 0.3120  (0.2330, 0.4150)  [0.0355, 0.5450] |
|  | *Η* | 3.23x10^-9^  (2.31x10^-9^,1.37 x10^-8^)  [1.49x10^-9^,2.52 x10^-7^] | 9.32 x10^-9^  (3.19 x10^-9^, 1.67x10^-7^) [2.45 x10^-9^, 5.21x10^-3^] | 6.50 x10^-9^  (3.63 x10^-9^, 6.08 x10^-8^)  [2.38 x10^-9^, 1.12x10^-4^] |
|  | *Incubation Period* | 6.18  (5.69, 6.86)  [4.88, 8.07] | 6.11  (5.22, 6.71)  [1.09, 8.87] | 6.02  (5.11, 6.68)  [1.93, 7.08] |
| A/γ=108/ml  Log-likelihood=  -964 | *β(x10^-11^)* | 3.67  (3.40, 3.94) | 4.62  (4.48, 4.73) | 5.50  (5.34, 5.67) |
|  | *δ* | 3.50  (3.29, 3.74) | 5.24  (5.07, 5.32) | 6.32  (6.07, 6.48) |
|  | *z_0_* | 0.397  (0.335, 0.471)  [0.240, 0.571] | 0.375  (0.319, 0.450)  [0.0809, 0.679] | 0.401  (0.358, 0.452)  [0.142, 0.578] |
|  | *Η* | 2.96x10^-9^  (1.68x10^-9^, 1.12x10^-8^)  [1.83x10^-10^,2.93x10^-7^] | 1.07x10^-9^  (1.16x10^-8^, 2.09x10^-7^)  [2.85x10^-10^, 4.47x10^-3^] | 5.57x10^-10^  (2.27x10^-9^, 4.56x10^-8^)  [2.78x10^-10^, 1.06x10^-4^] |
|  | *Incubation Period* | 6.08  (5.64, 6.47)  [4.70, 10.5] | 6.00  (5.18, 6.99)  [1.23, 9.24] | 5.99  (4.91, 7.09)  [1.28, 8.01] |

**Supplementary Figure S2a**

Fit of model variant in which the immune response clears free virus (with *z_0_, η, IP* fitted as patient-specific and *β,* *δ * fitted as group level) fitted to all patients, assuming *A=*1.4x10^6^/ml/day. Model variant shown in Supplementary Information Equations S1, parameter values given in Supplementary Table S2 Results shown for representative selected patients, all outputs on a log scale. Viremia data points shown as black dots (filled: above detection limit; unfilled: below the limit of detection). Black lines: fitted median viral dynamics, Grey lines: sample from posterior for virus dynamics, Pink lines: sample from posterior for immune response dynamics, Purple lines: sample from posterior of target (uninfected) cell dynamics. First row: primary DF patients; second row: secondary DF patients; third row secondary DHF patients.


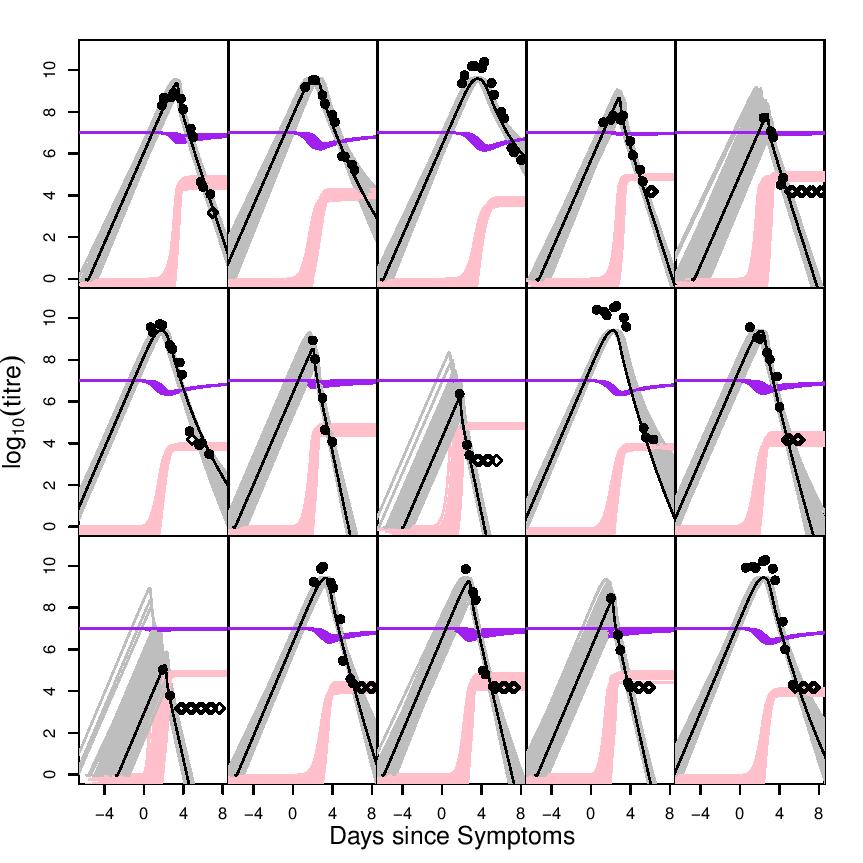


**Supplementary Figure S2b**

Fit of model variant in which the immune response clears free virus (with *z_0_, η, IP* fitted as patient-specific and *β,* *δ * fitted as group level) fitted to all patients, assuming *A=*1.4x10^7^/ml/day. Model variant shown in Supplementary Information Equations S1, parameter values given in Supplementary Table S2. Results shown for representative selected patients, all outputs on a log scale. Viremia data points shown as black dots (filled: above detection limit; unfilled: below the limit of detection). Black lines: fitted median viral dynamics, Grey lines: sample from posterior for virus dynamics, Pink lines: sample from posterior for immune response dynamics, Purple lines: sample from posterior of target (uninfected) cell dynamics. First row: primary DF patients; second row: secondary DF patients; third row secondary DHF patients.

**
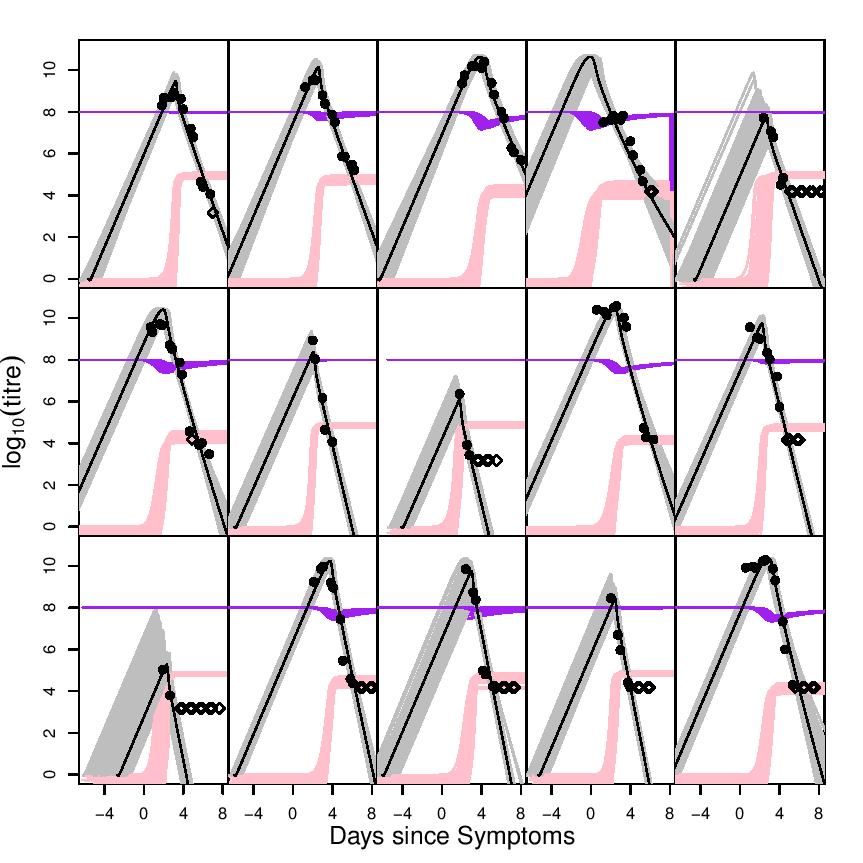
**

**Supplementary Table S4**

Parameter estimates for the model assuming that the target cells are not regenerated if depleted during infection , i.e. γ=0.00014. Target cell density (*A*/γ) is as before assumed to be 10^7^/ml. Patient specific parameter estimates are summarized with the median of median parameter estimates for each individual, inter-quartile range (in curved parentheses) and maximum/minimum (in square parentheses) obtained across all fitted patients. Medians and 95% credible intervals are shown for the common and group specific parameters. Parameters *κ, ω* are fixed, *β, δ*  fitted as group-specific, *η, IP* and *z_0_* as patient-specific. Model fit shown in Supplementary Information Figure S2.

| **Model** | **Parameter** | **Primary DF** | **Secondary DF** | **Secondary DHF** |
| --- | --- | --- | --- | --- |
| *κ, β* group specific  *z_0_, η, IP* patient specific  (model 4) | *β(x10^-10^)* | 1.69  (1.44, 1.93) | 2.23  (2.05, 2.32) | 2.48  (2.24, 2.80) |
|  | *κ* | 3.39  (3.23 3.57) | 4.66  (4.62, 5.00) | 5.14  (4.91, 5.40) |
|  | *z_0_* | 0.375  (0.314, 0.389)  [0.270, 0.536] | 0.380  (0.192, 0.687)  [0.00175 0.957] | 0.375  (0.341, 0.416)  [0.162, 0.556] |
|  | *η* | 1.37x10^-5^  (9.42x10^-6^ 6.42x10^-5^)  [7.10x10^-7^, 1.15x10^-3^] | 3.36 x10^-5^  (1.90x10^-6^, 4.76x10^-4^) [3.64 x10^-7^, 7.01] | 7.23x10^-6^  1.87 x10^-6^ 1.48 x10^-4^  [4.71 x10^-7^, 0.269] |
|  | *Incubation Period* | 5.92  (5.53, 6.33)  [4.88, 6.72] | 5.47  (4.58, 6.54)  [1.80, 11.10] | 5.48  (4.50, 6.59)  [1.98, 7.62] |

**Supplementary Figure S3**

Fit of model variant in which the immune response clears infected cells (with *z_0_, η, IP* fitted as patient-specific and *β,* *δ * fitted as group specific) fitted to all patients, in which the target cells are not regenerated if depleted during infection, γ=0.00014, target cell density remains as before (*A*/γ) of 10^7^/ml. Results shown for representative selected patients, all outputs on a log scale. Viremia data points shown as black dots (filled: above detection limit; unfilled: below the limit of detection). Black lines: fitted median viral dynamics, Grey lines: sample from posterior for virus dynamics, Pink lines: sample from posterior for immune response dynamics, Purple lines: sample from posterior of target (uninfected) cell dynamics. First row: primary DF patients; second row: secondary DF patients; third row secondary DHF patients. Estimated model parameter shown in Supplementary Table S3.


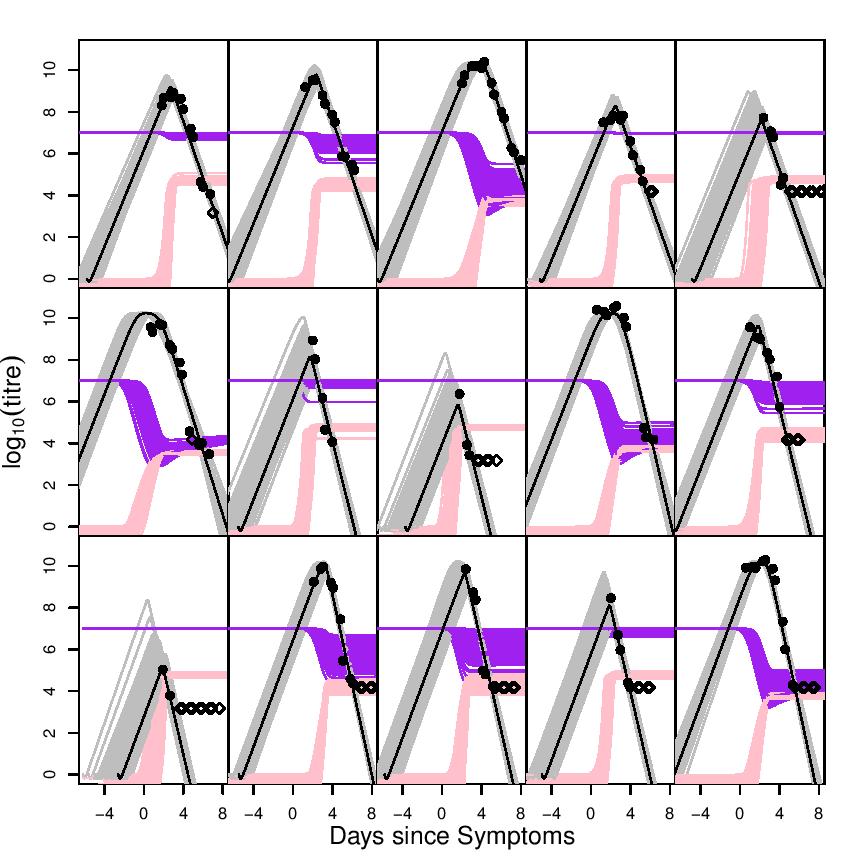

Supplement: Supplementary Information [file rsif20140094supp1.doc]
